# Lasting increases in trait mindfulness after psilocybin correlate positively with the mystical-type experience in healthy individuals

**DOI:** 10.3389/fpsyg.2022.948729

**Published:** 2022-10-05

**Authors:** Anna Søndergaard, Martin Korsbak Madsen, Brice Ozenne, Sophia Armand, Gitte Moos Knudsen, Patrick MacDonald Fisher, Dea Siggaard Stenbæk

**Affiliations:** ^1^Neurobiology Research Unit and NeuroPharm, Copenhagen University Hospital Rigshospitalet, Copenhagen, Denmark; ^2^Institute of Clinical Medicine, University of Copenhagen, Copenhagen, Denmark; ^3^Department of Psychiatry, University Hospital Svendborg, Svendborg, Denmark; ^4^Section of Biostatistics, Department of Public Health, University of Copenhagen, Copenhagen, Denmark; ^5^Institute of Psychology, University of Copenhagen, Copenhagen, Denmark

**Keywords:** psilocybin, mindfulness, psychedelics, mystical experience, serotonin 2A receptor, [^11^C]-Cimbi-36, Mindful Attention and Awareness Scale

## Abstract

**Background:**

Psilocybin-induced mystical-type experiences are associated with lasting positive psychological outcomes. Recent studies indicate that trait mindfulness is increased 3 months after psilocybin intake, preceded by decreases in neocortical serotonin 2A receptor (5-HT_2A_R) binding. However, the association between psilocybin-induced mystical-type experiences and subsequent changes in trait mindfulness remains unexplored, as does the association between pre-drug trait mindfulness and 5-HT_2A_R binding in the healthy brain.

**Aim:**

We evaluated whether psilocybin induced lasting increases in trait mindfulness in healthy volunteers, and whether the mystical-type experience was associated with this increase. We further examined the association between pre-drug trait mindfulness and 5-HT_2A_R binding in neocortex and selected frontolimbic regions.

**Materials and methods:**

Forty-six medium-high dose psilocybin sessions were conducted in 39 healthy individuals. The mystical-type experience was measured with the Mystical Experience Questionnaire (MEQ) at the end of the session. Trait mindfulness was measured using the Mindful Attention and Awareness Scale (MAAS) at baseline and 3 months after the psilocybin session. Thirty-two of the participants completed pre-drug [^11^C]-Cimbi-36 positron emission tomography (PET) to assess 5-HT_2A_R binding in neocortex and, *post-hoc*, in the frontolimbic regions amygdala, frontal cortex, and anterior cingulate cortex.

**Results:**

The MAAS score was significantly increased at 3-month follow-up (*p* = 3.24 × 10^–6^), a change positively associated with the MEQ score (*p* = 0.035). Although the association between pre-drug MAAS score and neocortex 5-HT_2A_R binding was not significant (*p* = 0.24), *post-hoc* analyses revealed a significant negative association between MAAS and right amygdala 5-HT_2A_R binding (p_FWER_ = 0.008).

**Conclusion:**

We here show that lasting changes in trait mindfulness following psilocybin administration are positively associated with intensity of the mystical-type experience, suggesting that the acute phenomenology of psilocybin facilitates a shift in awareness conducive for mindful living. We furthermore show that higher pre-drug trait mindfulness is associated with reduced 5-HT_2A_R binding in the right amygdala.

## Introduction

Psilocybin is a tryptamine alkaloid, naturally present in the *Psilocybe* genus of mushrooms, currently being researched as a promising therapeutic agent for a range of psychiatric disorders (Carhart-Harris and Goodwin, 2017; Andersen et al., 2021). In clinical studies, psilocybin shows immediate and sustained effects on depression (Carhart-Harris et al., 2016, 2018, 2021; Davis et al., 2021), obsessive-compulsive disorder (Moreno et al., 2006), anxiety and depression in terminal cancer patients (Grob et al., 2011; Griffiths et al., 2016; Ross et al., 2016), alcohol abuse (Bogenschutz et al., 2015; Garcia-Romeu et al., 2019) and smoking (Johnson et al., 2014, 2017). With increasing evidence in favor of positive effects of psilocybin, it is crucial to explore potential associated psychological factors. A small (*n* = 10) study has recently demonstrated increases in trait mindfulness in healthy individuals 3 months following a psilocybin session with no related mindfulness-training (Madsen et al., 2020). Similarly, significant increases in trait mindfulness have been observed as soon as 24 h after psilocybin intake in experienced meditators compared to placebo (Smigielski et al., 2019a). Trait mindfulness can be defined as *“the awareness that emerges through paying attention on purpose, in the present moment, and non-judgmentally to the unfolding of experience moment by moment.”* (Kabat-Zinn, 2003, p. 145). It is positively associated with trait openness (Giluk, 2009), improved behavioral regulation (Keng et al., 2011) and positive bias (Kiken and Shook, 2011), and negatively associated with trait neuroticism (Giluk, 2009), emotional reactivity (Baer et al., 2006) and negative bias (Kiken and Shook, 2011), suggesting that higher trait mindfulness is an indicator of greater psychological health. Several techniques to evoke a state of mindfulness stem from Buddhist meditation (Kabat-Zinn, 2003; Bishop et al., 2004), and have been integrated as components of third-wave cognitive behavior therapy, such as Acceptance and Commitment Therapy (ACT) (Hayes et al., 2006), Mindfulness Based Cognitive Therapy (MBCT) (Segal et al., 2002) and Mindfulness Based Stress Reduction (MBSR) (Kabat-Zinn, 1990), so called mindfulness-based interventions (MBI’s). Several researchers have postulated MBI as a framework for psychedelic drug administration due to similar phenomenology and psychological benefits that may act synergistically in combination (Heuschkel and Kuypers, 2020; Sloshower et al., 2020; Eleftheriou and Thomas, 2021). However, more empirical data is needed to gain a better understanding of how acute psychoactive effects of psilocybin are potentially associated with changes in trait mindfulness.

The psychoactive metabolite of psilocybin, psilocin, dose-dependently activates the serotonin 2A receptor (5-HT_2A_R) (Vollenweider et al., 1998; Nichols, 2016; Madsen et al., 2019), alters cerebral functional network changes (Carhart-Harris et al., 2012; Madsen et al., 2021) and induces an altered state of consciousness characterized by three phases: ascent, peak and descent, lasting 4–6 h (Griffiths et al., 2011; Madsen et al., 2019; Stenbæk et al., 2020). The psilocybin-induced psychedelic experience has a unique phenomenology, including effects such as altered visual and auditory perception, audio-visual synesthesia, enhanced emotions and meaning-making and changes in sense of self (Preller and Vollenweider, 2018). Interestingly, when administered in medium-to-high doses (>12 mg), psilocybin can induce a highly meaningful experience (Griffiths et al., 2006, 2008) known as the mystical-type experience, characterized as an experience of unity with all that exists, a sense of awareness of the fundamental truths of reality, deepfelt blissful mood, transcendence of space and time and difficulty describing the experience with words, termed ineffability (Stace, 1960; Pahnke, 1963; Barrett and Griffiths, 2018). Having a mystical-type experience during psilocybin administration has been associated with persistent positive effects (McCulloch et al., 2022) and increases in trait openness (Maclean et al., 2011) in healthy volunteers, as well as improvements in symptoms in psychiatric patient populations (Bogenschutz et al., 2015; Carhart-Harris et al., 2016; Griffiths et al., 2016; Ross et al., 2016; Roseman et al., 2018; Garcia-Romeu et al., 2019). The psilocybin-induced mystical-type experience shares many phenomenological features with mindful states, including altered self-referential processing (Smigielski et al., 2019b). It is therefore possible that having a mystical experience in the context of psilocybin administration could be a catalyst for lasting changes in trait mindfulness.

We have recently reported a significant negative association between changes in neocortical 5-HT_2A_R binding 1 week after psilocybin administration and changes in trait mindfulness after 3 months (Madsen et al., 2020), suggesting an involvement of 5-HT_2A_R in trait mindfulness. We also found that lower pre-drug 5-HT_2A_R binding, i.e., unstimulated by psilocybin, predicted the temporal unfolding of psychoactive effects of psilocybin, including greater intensity of the mystical-type experience (Stenbæk et al., 2020). 5-HT_2A_R binding in selected frontolimbic brain regions has previously been positively associated to trait neuroticism (Frokjaer et al., 2008), a trait inversely related to trait mindfulness (Brown and Ryan, 2003; Giluk, 2009; Jensen et al., 2016). Given that 5-HT_2A_R is critical for the described mind-expanding effects of psilocybin (Vollenweider et al., 1998; Stenbæk et al., 2020), it is conceivable that 5-HT_2A_R may be involved in mindful and meditative states that are often also described as expanded states (Hölzel et al., 2011; Smigielski et al., 2019b). However, the association between pre-drug 5-HT_2A_R binding and trait mindfulness has not yet been investigated.

In the present study, we evaluate the effect of psilocybin on changes in trait mindfulness from baseline to 3-month follow-up, and whether the mystical-type experience during acute psilocybin administration is associated with these changes in trait mindfulness. Lastly, we explore whether pre-drug 5-HT_2A_R binding in neocortex and frontolimbic regions is associated with trait mindfulness. We hypothesize that: (1) Trait mindfulness will increase from baseline to 3-month following psilocybin administration, (2) The intensity of the mystical-type experience is positively associated with changes in trait mindfulness, and (3) Pre-drug 5-HT_2A_R binding in neocortex and frontolimbic regions is negatively associated with trait mindfulness.

## Materials and methods

### Participants

The study included 39 participants who received a psychoactive psilocybin dose (>12 mg) and completed baseline and 3-month follow-up measures of trait mindfulness, as well as a post-session measure of the mystical-type experience. Of these, seven participants completed two psilocybin interventions with new baseline and follow-up measures, at least 12 months apart (mean months between interventions (SD) [range]: 21 (5) [12–28]). Thus, in order to increase power for the analyses, a total of 46 datasets were included in the analyses pertaining to hypotheses (1) and (2). For the analyses pertaining to hypothesis (3), 32 of the included participants also completed a pre-drug Positron Emission Tomography (PET) scan with the tracer [^11^C]-Cimbi-36 for imaging of 5-HT_2A_R binding in neocortex and frontolimbic regions (Ettrup et al., 2014, 2016).

All participants were recruited from a database of individuals volunteering to participate in human neuroimaging studies of psilocybin. Exclusion criteria included (a) present or previous psychiatric disorder in participant or immediate family, (b) present or previous neurological illness, severe somatic illness, or present medication that could affect the results, (c) non-fluency in Danish, vision or hearing impairment, (d) Present or previous learning disabilities, (e) current pregnancy or breastfeeding for women, (f) contraindications for MR-imaging, (g) alcohol or drug abuse, (h) allergy to the test drugs, (i) significant exposure to radioactivity within the past year, e.g., due to medical imaging, (j) ECG indicative of cardiac disease or use of medication causing prolonged QT-interval, (k) previous negative side-effects from hallucinogens, (l) use of psychedelics in the past 6 months, (m) blood donation less than 3 months before project participation, (n) hemoglobin <7.8 mM for women and 8.4 mM for men, (o) low plasma ferritin (<12 μg/L) and (p) bodyweight <50 kg. This was ensured through a complete physical and neurological exam, including ECG and blood screening for pathology, and a psychiatric screening, using the Mini International Neuropsychiatric Interview (Sheehan et al., 1998).

### Experimental procedures

#### Psilocybin interventions

Prior to the intervention day, participants met with the psychological support staff (one trained lead psychologist and one psychology trainee) to prepare for the psilocybin intervention, which included being informed about potential side-effects and safety precautions. On the intervention day, psilocybin was administered orally in 3 mg capsules with a glass of water based on a maximum weight-adjusted dose of 0.21 mg/kg (*n* = 12 sessions) or 0.31 mg/kg (*n* = 34 sessions). Of the 46 psilocybin interventions, 6 took place partly in a PET-scanner during acute effects (Madsen et al., 2019); 18 took place in a comfortable and private room (Madsen et al., 2020), and 22 took place partly in a MR-scanner during acute effects (Madsen et al., 2021). The psychological support staff members were present with the participant throughout the intervention day to provide interpersonal support. All participants met with the psychological support staff the day after intervention for an integration session. All preparation and integration sessions were standardized across interventions and were conducted by the same lead psychologist.

### Outcome measures

#### Mindful Attention and Awareness Scale

We used the Danish version of the Mindful Attention and Awareness Scale (MAAS) (Jensen et al., 2016) to assess the participants’ trait mindfulness at baseline and 3 months after the psilocybin intervention. The MAAS is a self-report scale, comprising 15 items related to general tendencies of inattentiveness toward emotions, thought, activities and physical sensations in the everyday experience, rated on a six-point Likert scale from one (almost always) to six (almost never). Example items include *“I find it difficult to stay focused on what’s happening in the present”* and *“I find myself preoccupied with the future or the past.”* The total MAAS score is calculated as the mean of all 15 items. Higher scores indicate higher degrees of trait mindfulness. The MAAS has a unidimensional construct with excellent internal consistency (Cronbach’s alpha for this dataset: baseline α = 0.86, follow-up α = 0.90) and good test-rest reliability (Brown and Ryan, 2003; Jensen et al., 2016).

#### Mystical Experience Questionnaire

Approximately 6 h after psilocybin administration on the intervention day, participants completed the Danish version of the 30-item version of the Mystical Experience Questionnaire (MEQ) (Barrett et al., 2015). The MEQ is a self-report scale to assess the intensity of mystical experience related to a discrete event, such as during acute psilocybin effects. Participants rated each item on a 6-point Likert scale [0 = none at all, 5 = extreme (more than ever before in my life and stronger than 4)] on four subscales: mystical (e.g., *“freedom from the limitations of your personal self and feeling of unity or bond with what was felt to be greater than your personal self”*), positive mood (e.g., *“experience of ecstasy”*), transcendence of time and space (e.g., *“loss of your usual sense of time”*) and ineffability (e.g., *“feeling that you could not do justice to your experience by describing it in words”)*. The total MEQ score is calculated as the mean of all items.

#### Positron Emission Tomography and Magnetic Resonance Imaging

Thirty-two participants were scanned at baseline using High Resolution Research Tomography PET scanner (CTI/Siemens, Knoxville, USA) with an approximate in-plane resolution of 2 mm for 120 min after a bolus injection of [^11^C]-Cimbi-36 tracer to reflect and image 5-HT_2A_R binding (Ettrup et al., 2014, 2016). The scans were reconstructed into 45 frames (6 × 10 s, 6 × 20 s, 6 × 60 s, 8 × 120 s, 19 × 300 s). For the purpose of PET-image co-registration and segmentation, high resolution 3D T1-weighted and T2-weighted images were acquired on a 3T prisma magnetic resonance imaging (MRI) Scanner (Siemens, Erlangen, Germany), using either a 64-channel or a 32-channel head coil. Regional time-activity curves including cerebellum as a reference region were extracted, as previously described (Svarer et al., 2005), and kinetic modeling was done using the simplified reference tissue model (SRTM) to compute the regional non-displaceable binding potential (BP_ND_) (Ettrup et al., 2014, 2016). Neocortical (a volume-weighted average of all cortical regions) [^11^C]-Cimbi-36 BP_ND_ was chosen as our primary outcome region based on findings from our previous study (Madsen et al., 2020), the high expression of 5-HT_2A_R within neocortex (Beliveau et al., 2017) and the high degree of inter-regional correlation across neocortical subregions (Erritzoe et al., 2010; Spies et al., 2020). As secondary outcomes for *post-hoc* analyses, [^11^C]-Cimbi-36 BP_ND_ in frontolimbic regions [frontal cortex, left and right amygdala and left and right anterior cingulate cortex (ACC)] were chosen based on a previous study (Armand et al., 2022).

### Data analysis

#### Psilocybin, Mystical Experience Questionnaire, and change in Mindful Attention and Awareness Scale

To evaluate the change in MAAS score from baseline to 3-month follow-up after psilocybin administration (Hypothesis 1) and its association with MEQ total score (Hypothesis 2), we used a linear mixed-effect model (LMM) for each hypothesis, as this model accounts for the repeated measures. To account for the seven participants who had completed two psilocybin interventions, participant-ID was included as a random effect. Baseline MAAS score was included as a covariate for Hypothesis 2, as magnitude of the change naturally is dependent on the baseline MAAS score due to the upper limit of the scale being six, and baseline MAAS has been associated to change in MAAS following a mindfulness-intervention (Shapiro et al., 2011). Although psilocybin dose and setting (Studerus et al., 2012) have been associated with acute psychedelic effects and positive psychological outcomes, inclusion of these as covariates in our model did not substantially affect the results, and were therefore not included in the final analyses. We report change in MAAS as the percentage change with a 95% confidence interval.

#### *Post-hoc* analysis of Mystical Experience Questionnaire subscales and change in Mindful Attention and Awareness Scale

In *post-hoc* analyses, we examined associations between change in MAAS and the individual MEQ subscales mystical, positive mood, transcendence of time and space, and ineffability, using the same LMM and under the same conditions presented in section “Psilocybin, Mystical Experience Questionnaire, and change in Mindful Attention and Awareness Scale.”

#### Pre-drug neocortical [^11^C]-Cimbi-36 binding and Mindful Attention and Awareness Scale

To evaluate the association between pre-drug neocortical [^11^C]-Cimbi-36 BP_ND_ and MAAS score, (Hypothesis 3) we fit a linear regression model with [^11^C]-Cimbi-36 BP_ND_ as independent variable and MAAS score as the dependent variable. Given that MAAS score is associated to age (Jensen et al., 2016) and body mass index (BMI) (Camilleri et al., 2015), these were included as covariates. Effects for the models are reported as unstandardized regression coefficients (β) with 95% confidence intervals (95% CI) and adjusted *R*^2^ as a measure of variance.

#### *Post-hoc* analyses of pre-drug frontolimbic [^11^C]-Cimbi-36 binding and Mindful Attention and Awareness Scale

In *post-hoc* analyses, we evaluated the associations between pre-drug [^11^C]-Cimbi-36 BP_ND_ in the frontolimbic brain regions (total frontal cortex and right and left amygdala and ACC) and MAAS score. Covariates and procedures for reporting effects for the models were identical to those presented in section “Pre-drug neocortical [^11^C]-Cimbi-36 binding and Mindful Attention and Awareness Scale.”

#### Statistical significance and effect size

For Hypothesis 1, we conducted two significance tests: one with (*n* = 46) and one without the 10 participants from our previous study (*n* = 36) (Madsen et al., 2020), to demonstrate an independent replication. Cohen’s d is reported as an expression of standardized effect size (Cohen, 1988) for Hypothesis 1. The threshold for statistical significance for Hypothesis 1, 2 and 3 was *p* < 0.05. For the *post-hoc* analyses, *p*-values were corrected for multiple tests using the Bonferroni method; four tests for the *post-hoc* analyses regarding the MEQ subscales, and five tests for the *post-hoc* analyses regarding pre-drug [^11^C]-Cimbi-36 BP_ND_ in frontolimbic regions. For *post-hoc* analyses, *p*-values are reported both uncorrected (p_unc_) and with a family-wise error rate correction (p_FWER_) using a statistical threshold of p_FWER_ < 0.05. All statistical analyses were conducted using the statistical software *R (v4.0.5)*.

## Results

### Participant characteristics and descriptive statistics

Participant characteristics and descriptive data are summarized in Table 1. Previous psychedelic use covers prior experience with 5-HT_2A_R agonistic psychedelics. A figure graphically displaying the change in each MAAS item score from baseline to 3-month follow-up can be found in Supplementary Figure 1.

**TABLE 1:** Participant characteristics and descriptive statistics.

Variables	Mean ± SD	Median	Range: min, max
**Interventions *n* = 46**			
Sex (% female)	37		
Previous psychedelic use (% yes)	37		
Age (years)	32.6 ± 8.69	29.8	24.2, 60.2
BMI (kg/m^2^)*	24.5 ± 3.09	23.8	18.8, 33.2
Weight-adjusted psilocybin dose (mg/kg)	0.26 ± 0.04	0.27	0.15, 0.32
Actual psilocybin dose (mg)	20.2 ± 4.36	21	12, 30
Time from psilocybin intervention to follow-up MAAS score (months)	3.21 ± 0.55	3.10	2.55, 5.35
Baseline MAAS score	4.14 ± 0.6	4.2	2.33, 5.13
Neocortical [^11^C]-Cimbi-36 BP_ND_**	1.21 ± 0.220	1.17	0.870, 2.02

*For the participants that participated in two interventions, only BMI from the first intervention is included (*n* = 39), as only PET data from the first intervention was included in Hypothesis 3, where BMI was used as a covariate. **n = 32.

### Changes in Mindful Attention and Awareness Scale following psilocybin administration

**FIGURE 1:**
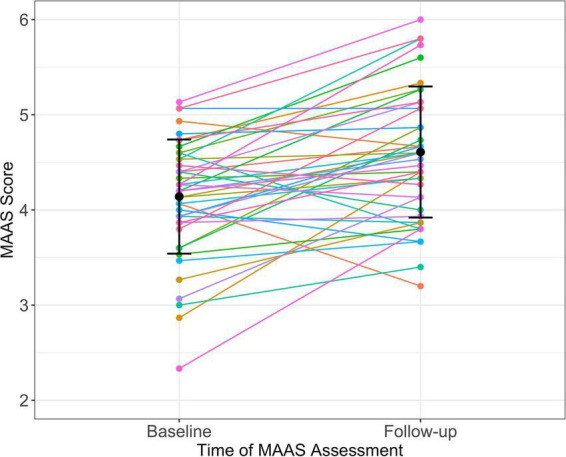
Change in Mindful Attention and Awareness Scale (MAAS) score following psilocybin administration from baseline to 3-month follow-up in the full sample (*n* = 46). Colored lines: Individual values; middle black dot: mean; error bar: SD. No participants had a total MAAS score <2. Maximum possible MAAS score = 6.

We found a significant increase in average MAAS score from baseline to 3-month follow-up, both in the full sample (*n* = 46) (Cohen’s d: 0.72; mean% change [95%CI]: 8.1 [5.1;11.1]; *p* = 3.24 × 10^–6^, Figure 1) and in the independent sample (*n* = 36) (Cohen’s d: 0.71; mean% change [95%CI]: 7.5 [4.0;10.9]; *p* = 1.0 × 10^–4^). A graphical display of relation between baseline MAAS and change in MAAS is illustrated in Supplementary Figure 2.

### Mystical Experience Questionnaire and change in Mindful Attention and Awareness Scale

**FIGURE 2:**
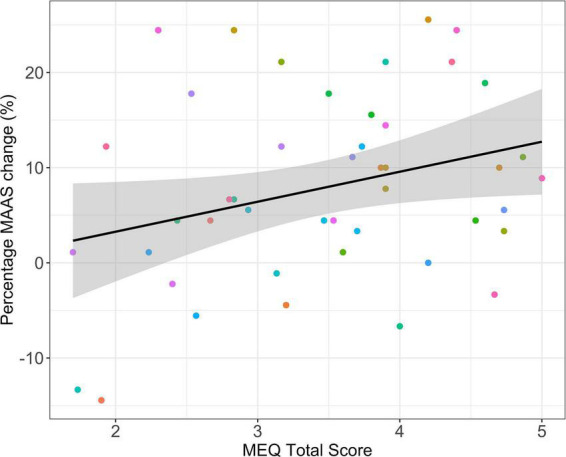
Mystical Experience Questionnaire (MEQ) total score association with percentage change in Mindful Attention and Awareness Scale (MAAS). Black line and gray shading: Estimated regression line and 95% confidence interval. Colored circles: Individual participants. No participants had an MEQ score ≤1.

MEQ total score was significantly positively associated with change in MAAS score (% mean change per unit increase in MEQ [95%CI]: 3.1 [0.04; 6.0]; *p* = 0.035, Figure 2).

### *Post-hoc*: Mystical Experience Questionnaire subscales and changes in Mindful Attention and Awareness Scale

*Post-hoc* analyses revealed a statistically significant positive association between change in MAAS score and the MEQ subscale mystical (% mean change per unit increase in mystical [95%CI]: 2.93 [0.75; 5.11]; p_unc_ = 0.012, p_FWER_ = 0.049). No other significant associations between change in MAAS score and MEQ subscales were observed after correction for multiple testing (positive mood: 3.40 [0.67; 6.13]; p_unc_ = 0.019, p_FWER_ = 0.076, transcendence of time and space: 0.48 [−2.57; 3.57]; p_unc_ = 0.75, p_FWER_ = 3 and ineffability: −0.13 [−3.01; 2.76]; p_unc_ = 0.93, p_FWER_ = 3.72).

### Pre-drug neocortical [^11^C]-Cimbi-36 BP_ND_ and Mindful Attention and Awareness Scale

The linear regression model showed a negative but not significant association between pre-drug neocortical [^11^C]-Cimbi-36 BP_ND_ and MAAS score (β [95%CI], *R*^2^: −0.55 [−1.48; 0.38], 0.0051; *p* = 0.24).

### *Post-hoc*: Pre-drug frontolimbic [^11^C]-Cimbi-36 BP_ND_ and Mindful Attention and Awareness Scale

**FIGURE 3:**
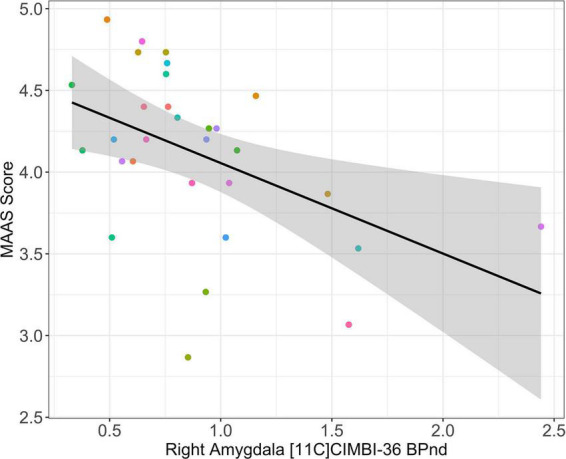
Pre-drug right amygdala [^11^C]-Cimbi-36 BP_ND_ association with Mindful Attention and Awareness Scale (MAAS) score. Black line and gray shading: Estimated regression line and 95% confidence interval. Colored circles: Individual participants. No participants had a MAAS score <2 or >5. Maximum possible MAAS score = 6.

*Post-hoc* analyses revealed a significant negative association between pre-drug right amygdala [^11^C]-Cimbi-36 BP_ND_ and MAAS score (β [95%CI], *R*^2^: −0.67 [–1.06; −0.28], 0.30; p_unc_ = 0.0016, p_FWER_ = 0.008, Figure 3). No other significant associations between [^11^C]-Cimbi-36 BP_ND_ in frontolimbic regions and MAAS score were observed after correction for multiple testing (frontal cortex: −0.48 [−1.35; 0.38], 0.00015; p_unc_ = 0.26, p_FWER_ = 1.3), right ACC: −0.19 [−1.54; 0.17], −0.0047; p_unc_ = 0.29, p_FWER_ = 1.45, left ACC: −0.31 [−0.90; −0.28], – 0.0056; p_unc_ = 0.30, p_FWER_ = 1.5 and left amygdala: (−0.14 [−1.71; −0.89], −0.05; p_unc_ = 0.78, p_FWER_ = 3.9).

## Discussion

We here demonstrate that psilocybin induces a significant increase in trait mindfulness of 8.1% from baseline to 3-month follow-up across 46 psilocybin sessions. As a novel finding, we show that participants who experienced a greater mystical-type experience exhibited a significantly greater increase in trait mindfulness at 3-month follow-up. We found no significant association between pre-drug neocortical 5-HT_2A_R binding and trait mindfulness; however, *post-hoc* analyses of frontolimbic brain regions revealed a significant negative association between pre-drug 5-HT_2A_R binding in the right amygdala and trait mindfulness.

The observed increase in trait mindfulness replicates and strengthens the results of our previous study, where psilocybin increased trait mindfulness for at least 3 months in 10 healthy volunteers (Madsen et al., 2020). These findings align with previous studies of psychedelic-induced mindfulness in healthy volunteers. Buddhist meditation practitioners show increased trait mindfulness the day after psilocybin compared to placebo (Smigielski et al., 2019a), and ayahuasca intake is associated with increased mindfulness 24 h after administration (Soler et al., 2016; Uthaug et al., 2018). More sustained effects have only been studied after 5-methoxy-N,N-dimethyltryptamine (5-MeO-DMT) intake where trait mindfulness was higher 1 month after intake (Uthaug et al., 2019). Taken together, there is a growing amount of evidence suggesting that psychedelic substances promote an immediate and sustained increase in trait mindfulness in healthy individuals.

Although trait mindfulness is considered a stable human disposition, it can be enhanced through meditation and mindfulness-practice (Smigielski et al., 2019a). Our findings of sustained and increased trait mindfulness following psilocybin administration can be interpreted in relation to a previous mindfulness-intervention, where healthy individuals participated in an 8-week intensive MBSR course and on average experienced a 7% increase in trait mindfulness, compared to 1–3% in the inactive control groups (Jensen et al., 2012), indicating a significant increase following MBSR training. Another MBSR study reported a sustained 8% increase in trait mindfulness over 12-months in the treatment group, compared to the control group where both increases and decreases in trait mindfulness were reported, fluctuating up to 7% in each direction (Shapiro et al., 2011). These results suggest that trait mindfulness naturally fluctuates over time, but on average at a lower magnitude and without a clear pattern compared to what is observed following MBSR treatment and psilocybin intervention, the latter as suggested by the results from the current study.

Our findings support a positive association between the intensity of the mystical-type experience and increases in trait mindfulness, echoing previous studies that have attested to the importance of the mystical-type experience for lasting positive psychological effects of psilocybin (Pahnke, 1963; Maclean et al., 2011; Bogenschutz et al., 2015; Carhart-Harris et al., 2016; Griffiths et al., 2016, 2018; Ross et al., 2016; Roseman et al., 2018; Garcia-Romeu et al., 2019; Smigielski et al., 2019a; McCulloch et al., 2022). In self-reported psilocybin accounts, healthy participants who have had a complete mystical-type experience (>60% on all subscales of the MEQ) describe feeling a universal bond, witnessing profound beauty and a great love for family (McCulloch et al., 2022). These themes of connectedness, bliss and unity with all things are also recounted in mindfulness practice, such as meditation (Hölzel and Ott, 2006; Barrett and Griffiths, 2018). Other notable parallels with meditation include the experience of ego-dissolution (Millière, 2017; Barrett and Griffiths, 2018; Millière et al., 2018) along with neurobiological similarities, such as reduced network integrity in the default mode network (Brewer et al., 2011; Carhart-Harris et al., 2014; Fox et al., 2016; Barrett and Griffiths, 2018). Furthermore, it has recently been demonstrated that both trait mindfulness and mystical-type experiences are associated with greater mental wellbeing, and that specifically psilocybin-induced mystical-type experiences alongside a mindfulness practice are associated with higher mindfulness and greater mental wellbeing (Qiu and Minda, 2022). It is possible that the subjective experience of merging with a “oneness” of all things together with a diminished focus on ego, at least when combined with a blissful or ecstatic emotional tone, allows for a shift in perspective that is conducive for psychological flexibility and mindful living. This is further supported by our post hoc finding of a significant positive association between the MEQ subscale “mystical” and change in trait mindfulness. Based on these initial findings, we encourage future research to study the possible complimentary effects of mindfulness-practice and psilocybin in clinical populations, as it may be a suitable therapeutic framework to maintain positive psychological effects of psilocybin treatment. It is also conceivable that intervention with psilocybin could be used to address barriers to MBI and potentially assist individuals who struggle with engagement in therapy.

In *post-hoc* analyses, we found a significant negative association between pre-drug 5-HT_2A_R binding in the right amygdala and trait mindfulness. There is some pre-clinical evidence to suggest an inverse coupling between available serotonin in the brain and 5-HT_2A_R binding (Jørgensen et al., 2018), further supported by the downregulation of 5-HT_2A_R binding following administration of serotonergic antidepressants (Sanders-Bush et al., 1989; Gray and Roth, 2001; Günther et al., 2009), also seen in depressed patients (Yatham et al., 1999). As such, it is possible that lower 5-HT_2A_R binding in the right amygdala reflects increased serotonin, and that this is coupled to higher trait mindfulness. However, interpretation of this finding should be made with caution, as the time-activity curve fit for amygdala is typically noisy (Finnema et al., 2014), and we observed no other significant associations for neocortex or frontolimbic regions, including the left amygdala. Interestingly, fMRI studies support involvement of the right amygdala in mindful states (Farb et al., 2007) and trait mindfulness (Way et al., 2010), suggesting that the right amygdala may be more involved in mindfulness than the left amygdala. Future studies are needed to replicate the observed association between right amygdala 5-HT_2A_R binding and trait mindfulness, and further explore the role of serotonin for individual differences in response to psychedelic administration.

### Limitations

Our findings should be interpreted in light of the following limitations: Since we did not collect data in the time that elapsed between psilocybin intervention and 3-months follow-up, we cannot determine whether follow-up MAAS scores were affected by other life circumstances during this time period. Furthermore, although MAAS has shown high test-retest reliability and is a stable personality trait measure of mindfulness (Brown and Ryan, 2003; Jensen et al., 2016), this study is limited by its open-label design and not having a blinded control group (see Muthukumaraswamy et al., 2021 for discussion).

### Conclusion

We demonstrate that trait mindfulness is increased in healthy volunteers at least 3 months following a single psilocybin intervention and that the self-reported mystical-type experience immediately following the psychedelic experience is positively associated with this lasting increase in trait mindfulness. We also show a negative association between pre-drug 5-HT_2A_R binding in the right amygdala and trait mindfulness. These findings suggest that psilocybin and the mystical-type experience could have mindfulness-enhancing capacities, and may potentially work in synergy with mindfulness-based forms of therapies in a clinical setting.

## Data availability statement

The datasets presented in this article are not readily available because of the General Data Protection Regulation (GDPR). However, data in the Cimbi database can be accessed by application (http://www.cimbi.dk/db). Requests to access the datasets should be directed to Peter S. Jensen, http://www.cimbi.dk/db.

## Ethics statement

The studies involving human participants were reviewed and approved by the Danish Medicines Agency (EudraCT ID: 2016-004000-61, amendments: 2017014166, 2017082837, and 2018023295); and the Ethics Committee for the Capital Region of Copenhagen (journal ID: H-16028698, with amendments 56023, 56967, 57974, 59673, 60437, 62255, 74340, and 79042). This study was conducted in accordance with the Declaration of Helsinki. The participants provided their written informed consent to participate in this study.

## Author contributions

AS collected the data, performed analyses, and wrote the manuscript. MM collected the data and provided feedback on the manuscript. BO provided statistical consultation. SA collected the data, assisted with the psilocybin interventions, and provided feedback on the manuscript. GK conceptualized the study, supervised the data collection, and provided feedback on the manuscript. PF collected the data, supervised the data collection, and provided feedback on the manuscript. DS conceptualized the study, assisted with the psilocybin interventions, and supervised the writing of the manuscript. All authors contributed to the article and approved the submitted version.

## References

[B1] Andersen K. A. Carhart-Harris R. Nutt D. J. Erritzoe D. (2021). Therapeutic effects of classic serotonergic psychedelics: A systematic review of modern-era clinical studies. *Acta Psychiatr. Scand. Scand.* 143 101–118. 10.1111/acps.13249 33125716

[B2] Armand S. Ozenne B. Svart N. Frokjaer V. G. Knudsen G. M. Fisher P. M. (2022). Brain serotonin transporter is associated with cognitive-affective biases in healthy individuals. *Hum. Brain Mapp.* 43 4174–4184. 10.1002/hbm.25946 35607850 PMC9374883

[B3] Baer R. A. Smith G. T. Hopkins J. Krietemeyer J. Toney L. (2006). Using self-report assessment methods to explore facets of mindfulness. *Assessment* 13 27–45. 10.1177/1073191105283504 16443717

[B4] Barrett F. S. Griffiths R. R. (2018). “Classic hallucinogens and mystical experiences: Phenomenology and neural correlates,” in *Behavioral neurobiology of psychedelic drugs. current topics in behavioral neurosciences*, eds Halberstadt A. L. Vollenweider F. X. Nichols D. E. (Berlin: Springer), 393–430. 10.1007/7854_2017_474 PMC6707356 28401522

[B5] Barrett F. S. Johnson M. W. Griffiths R. R. (2015). Validation of the revised Mystical Experience Questionnaire in experimental sessions with psilocybin. *J. Psychopharmacol.* 29 1182–1190. 10.1177/0269881115609019 26442957 PMC5203697

[B6] Beliveau V. Ganz M. Feng L. Ozenne B. Højgaard L. Fisher P. M. (2017). A high-resolution in vivo atlas of the human brain’s serotonin system. *J. Neurosci.* 37 120–128. 10.1523/JNEUROSCI.2830-16.2016 28053035 PMC5214625

[B7] Bishop S. R. Lau M. Shapiro S. Carlson L. Anderson N. D. Carmody J. (2004). Mindfulness: A proposed operational definition. *Clin. Psychol. Sci. Pract.* 11 230–241. 10.1093/clipsy/bph077

[B8] Bogenschutz M. P. Forcehimes A. A. Pommy J. Wilcox C. E. Barbosa P. C. R. Strassman R. J. (2015). Psilocybin-assisted treatment for alcohol dependence: A proof-of-concept study. *J. Psychopharmacol.* 29 289–299. 10.1177/0269881114565144 25586396

[B9] Brewer J. A. Worhunsky P. D. Gray J. R. Tang Y. Y. Weber J. Kober H. (2011). Meditation experience is associated with differences in default mode network activity and connectivity. *Proc. Natl. Acad. Sci. U.S.A.* 108 20254–20259. 10.1073/pnas.1112029108 22114193 PMC3250176

[B10] Brown K. W. Ryan R. M. (2003). The benefits of being present: Mindfulness and its role in psychological well-being. *J. Pers. Soc. Psychol.* 84 822–848. 10.1037/0022-3514.84.4.822 12703651

[B11] Camilleri G. M. Méjean C. Bellisle F. Hercberg S. Péneau S. (2015). Association between Mindfulness and weight status in a general population from the NutriNet-Santé study. *PLoS One* 10:e0127447. 10.1371/journal.pone.0127447 26038824 PMC4454654

[B12] Carhart-Harris R. L. Goodwin G. (2017). The therapeutic potential of psychedelic drugs: Past, present and future. *Neuropsychopharmacology* 42 2105–2113. 10.1038/npp.2017.84 28443617 PMC5603818

[B13] Carhart-Harris R. L. Bolstridge M. Day C. M. J. Rucker J. Watts R. (2018). Psilocybin with psychological support for treatment-resistant depression: Six-month follow-up. *Psychopharmacology (Berl.)* 235 399–408. 10.1007/s00213-017-4771-x 29119217 PMC5813086

[B14] Carhart-Harris R. L. Bolstridge M. Rucker J. Day C. M. J. Erritzoe D. Kaelen M. (2016). Psilocybin with psychological support for treatment-resistant depression: An open-label feasibility study. *Lancet Psychiatry* 3 619–627. 10.1016/S2215-0366(16)30065-7 27210031

[B15] Carhart-Harris R. L. Erritzoe D. Williams T. Stone J. M. Reed L. J. Colasanti A. (2012). Neural correlates of the psychedelic state as determined by fMRI studies with psilocybin. *Proc. Natl. Acad. Sci. U.S.A.* 109 2138–2143. 10.1073/pnas.1119598109 22308440 PMC3277566

[B16] Carhart-Harris R. L. Giribaldi B. Watts R. Baker-Jones M. Murphy-Beiner A. Murphy R. (2021). Trial of psilocybin versus escitalopram for depression. *N. Engl. J. Med.* 384 1402–1411. 10.1056/nejmoa2032994 33852780

[B17] Carhart-Harris R. L. Leech R. Hellyer P. J. Shanahan M. Feilding A. Tagliazucchi E. (2014). The entropic brain: A theory of conscious states informed by neuroimaging research with psychedelic drugs. *Front. Hum. Neurosci.* 8:20. 10.3389/fnhum.2014.00020 24550805 PMC3909994

[B18] Cohen J. (1988). *Statistical power analysis for the behavioral sciences*, 2nd Edn. Hillsdale, NJ: Lawrence Erlbaum Associates.

[B19] Davis A. K. Barrett F. S. May D. G. Cosimano M. P. Sepeda N. D. Johnson M. W. (2021). Effects of psilocybin-assisted therapy on major depressive disorder: A randomized clinical trial. *JAMA Psychiatry* 78 481–489. 10.1001/jamapsychiatry.2020.3285 33146667 PMC7643046

[B20] Eleftheriou M. E. Thomas E. (2021). Examining the potential synergistic effects between mindfulness training and psychedelic-assisted therapy. *Front. Psychiatry* 12:707057. 10.3389/fpsyt.2021.707057 34456763 PMC8386240

[B21] Erritzoe D. Holst K. Frokjaer V. G. Licht C. L. Kalbitzer J. Nielsen F. Å (2010). A nonlinear relationship between cerebral serotonin transporter and 5HT-2A receptor binding: An in vivo molecular imaging study in humans. *J. Neurosci.* 30 3391–3397. 10.1523/JNEUROSCI.2852-09.2010 20203198 PMC6634111

[B22] Ettrup A. Da Cunha-Bang S. Mcmahon B. Lehel S. Dyssegaard A. Skibsted A. W. (2014). Serotonin 2A receptor agonist binding in the human brain with [11 C]Cimbi-36. *J. Cereb. Blood Flow Metab.* 34 1188–1196. 10.1038/jcbfm.2014.68 24780897 PMC4083382

[B23] Ettrup A. Svarer C. McMahon B. da Cunha-Bang S. Lehel S. Møller K. (2016). Serotonin 2A receptor agonist binding in the human brain with [11C]Cimbi-36: Test–retest reproducibility and head-to-head comparison with the antagonist [18F]altanserin. *Neuroimage* 130 167–174. 10.1016/j.neuroimage.2016.02.001 26876490

[B24] Farb N. A. S. Segal Z. V. Mayberg H. Bean J. McKeon D. Zainab F. (2007). Attending to the present: Mindfulness meditation reveals distinct neural modes of self-reference. *Soc. Cogn. Affect. Neurosci.* 2 313–322. 10.1093/scan/nsm030 18985137 PMC2566754

[B25] Finnema S. J. Stepanov V. Ettrup A. Nakao R. Amini N. Svedberg M. (2014). Characterization of [11C]Cimbi-36 as an agonist PET radioligand for the 5-HT2A and 5-HT2C receptors in the nonhuman primate brain. *Neuroimage* 84 342–353. 10.1016/j.neuroimage.2013.08.035 23994452

[B26] Fox K. C. R. Dixon M. L. Nijeboer S. Girn M. Floman J. L. Lifshitz M. (2016). Functional neuroanatomy of meditation: A review and meta-analysis of 78 functional neuroimaging investigations. *Neurosci. Biobehav. Rev.* 65 208–228. 10.1016/j.neubiorev.2016.03.021 27032724

[B27] Frokjaer V. G. Mortensen E. L. Nielsen F.Å. Haugbol S. Pinborg L. H. Adams K. H. (2008). Frontolimbic serotonin 2A receptor binding in healthy subjects is associated with personality risk factors for affective disorder. *Biol. Psychiatry* 63, 569–576. 10.1016/j.biopsych.2007.07.009 17884017

[B28] Garcia-Romeu A. Davis A. K. Erowid F. Erowid E. Griffiths R. R. Johnson M. W. (2019). Cessation and reduction in alcohol consumption and misuse after psychedelic use. *J. Psychopharmacol.* 33 1088–1101. 10.1177/0269881119845793 31084460

[B29] Giluk T. L. (2009). Mindfulness, big five personality, and affect: A meta-analysis. *Pers. Individ. Dif.* 47 805–811. 10.1016/j.paid.2009.06.026

[B30] Gray J. A. Roth B. L. (2001). Paradoxical trafficking and regulation of 5-HT(2A) receptors by agonists and antagonists. *Brain Res. Bull.* 56 441–451. 10.1016/S0361-9230(01)00623-2 11750789

[B31] Griffiths R. R. Johnson M. W. Carducci M. A. Umbricht A. Richards W. A. Richards B. D. (2016). Psilocybin produces substantial and sustained decreases in depression and anxiety in patients with life-threatening cancer: A randomized double-blind trial. *J. Psychopharmacol.* 30 1181–1197. 10.1177/0269881116675513 27909165 PMC5367557

[B32] Griffiths R. R. Johnson M. W. Richards W. A. Richards B. D. Jesse R. MacLean K. A. (2018). Psilocybin-occasioned mystical-type experience in combination with meditation and other spiritual practices produces enduring positive changes in psychological functioning and in trait measures of prosocial attitudes and behaviors. *J. Psychopharmacol.* 32 49–69. 10.1177/0269881117731279 29020861 PMC5772431

[B33] Griffiths R. R. Johnson M. W. Richards W. A. Richards B. D. McCann U. Jesse R. (2011). Psilocybin occasioned mystical-type experiences: Immediate and persisting dose-related effects. *Psychopharmacology (Berl.)* 218 649–665. 10.1007/s00213-011-2358-5 21674151 PMC3308357

[B34] Griffiths R. R. Richards W. A. Johnson M. W. McCann U. D. Jesse R. (2008). Mystical-type experiences occasioned by psilocybin mediate the attribution of personal meaning and spiritual significance 14 months later. *J. Psychopharmacol.* 22 621–632. 10.1177/0269881108094300 18593735 PMC3050654

[B35] Griffiths R. R. Richards W. A. McCann U. Jesse R. (2006). Psilocybin can occasion mystical-type experiences having substantial and sustained personal meaning and spiritual significance. *Psychopharmacology (Berl.)* 187 268–283. 10.1007/s00213-006-0457-5 16826400

[B36] Grob C. S. Danforth A. L. Chopra G. S. Hagerty M. McKay C. R. Halberstadt A. L. (2011). Pilot study of psilocybin treatment for anxiety in patients with advanced-stage cancer. *Arch. Gen. Psychiatry* 68 71–78. 10.1001/archgenpsychiatry.2010.116 20819978

[B37] Günther L. Liebscher S. Jähkel M. Oehler J. (2009). Effects of chronic citalopram treatment on 5-HT1A and 5-HT2A receptors in group- and isolation-housed mice. *Eur. J. Pharmacol.* 593 49–61. 10.1016/j.ejphar.2008.07.011 18657534

[B38] Hayes S. C. Luoma J. B. Bond F. W. Masuda A. Lillis J. (2006). Acceptance and commitment therapy: Model, processes and outcomes. *Behav. Res. Ther.* 44 1–25. 10.1016/j.brat.2005.06.006 16300724

[B39] Heuschkel K. Kuypers K. P. C. (2020). Depression, mindfulness, and psilocybin: Possible complementary effects of mindfulness meditation and psilocybin in the treatment of depression. A review. *Front. Psychiatry* 11:224. 10.3389/fpsyt.2020.00224 32296353 PMC7136554

[B40] Hölzel B. K. Ott U. (2006). Relationships between meditation depth, absorption, meditation practice, and mindfulness: A latent variable approach. *J. Transpers. Psychol.* 38 179–199.

[B41] Hölzel B. K. Lazar S. W. Gard T. Schuman-Olivier Z. Vago D. R. Ott U. (2011). How does mindfulness meditation work? Proposing mechanisms of action from a conceptual and neural perspective. *Perspect. Psychol. Sci.* 6 537–559. 10.1177/1745691611419671 26168376

[B42] Jensen C. G. Niclasen J. Vangkilde S. A. Petersen A. Hasselbalch S. G. (2016). General inattentiveness is a long-term reliable trait independently predictive of psychological health: Danish validation studies of the mindful attention awareness scale. *Psychol. Assess.* 28 e70–e87. 10.1037/pas0000196 26751089

[B43] Jensen C. G. Vangkilde S. Frokjaer V. Hasselbalch S. G. (2012). Mindfulness training affects attention-Or is it attentional effort? *J. Exp. Psychol. Gen.* 141 106–123. 10.1037/a0024931 21910559

[B44] Johnson M. W. Garcia-Romeu A. Griffiths R. R. (2017). Long-term Follow-up of Psilocybin-facilitated Smoking Cessation. *Am. J. Drug Alcohol Abuse* 43 55–60. 10.3109/00952990.2016.1170135 27441452 PMC5641975

[B45] Johnson M. W. Garcia-Romeu A. Cosimano M. P. Griffiths R. R. (2014). Pilot study of the 5-HT2AR agonist psilocybin in the treatment of tobacco addiction. *J. Psychopharmacol.* 28 983–992. 10.1177/0269881114548296 25213996 PMC4286320

[B46] Jørgensen L. M. Weikop P. Villadsen J. Visnapuu T. Ettrup A. Hansen H. D. (2018). Cerebral 5-HT release correlates with [11C]Cimbi36 PET measures of 5-HT2A receptor occupancy in the pig brain. *J. Cereb. Blood Flow Metab.* 37 425–434. 10.1177/0271678X16629483 26825776 PMC5381441

[B47] Kabat-Zinn J. (1990). *Full catastrophe living: Using the wisdom of your mind to face stress, pain and illness.* New York, NY: Dell.

[B48] Kabat-Zinn J. (2003). Mindfulness-based interventions in context: Past, present, and future. *Clin. Psychol. Sci. Pract.* 10 144–156. 10.1093/clipsy/bpg016

[B49] Keng S.-L. Smoski M. J. Robins C. J. (2011). Effects of mindfulness on psychological health. *Clin. Psychol. Rev.* 31 1041–1056. 10.1016/j.cpr.2011.04.006 21802619 PMC3679190

[B50] Kiken L. G. Shook N. J. (2011). Looking up: Mindfulness increases positive judgments and reduces negativity bias. *Soc. Psychol. Personal. Sci.* 2 425–431. 10.1177/1948550610396585

[B51] Maclean K. A. Johnson M. W. Griffiths R. R. (2011). Mystical experiences occasioned by the hallucinogen psilocybin lead to increases in the personality domain of openness. *J. Psychoph.* 25 1453–1461. 10.1177/0269881111420188 21956378 PMC3537171

[B52] Madsen M. K. Fisher P. M. Burmester D. Dyssegaard A. Stenbæk D. S. Kristiansen S. (2019). Psychedelic effects of psilocybin correlate with serotonin 2A receptor occupancy and plasma psilocin levels. *Neuropsychopharmacology* 44 1328–1334. 10.1038/s41386-019-0324-9 30685771 PMC6785028

[B53] Madsen M. K. Fisher P. M. Stenbæk D. S. Kristiansen S. Burmester D. Lehel S. (2020). A single psilocybin dose is associated with long-term increased mindfulness, preceded by a proportional change in neocortical 5-HT2A receptor binding. *Eur. Neuropsychopharmacol.* 33 71–80. 10.1016/j.euroneuro.2020.02.001 32146028

[B54] Madsen M. K. Stenbæk D. S. Arvidsson A. Armand S. Marstrand-Joergensen M. R. Johansen S. S. (2021). Psilocybin-induced changes in brain network integrity and segregation correlate with plasma psilocin level and psychedelic experience. *Eur. Neuropsychopharmacol.* 50 121–132. 10.1016/j.euroneuro.2021.06.001 34246868

[B55] McCulloch D. E.-W. Grzywacz M. Z. Madsen M. K. Jensen P. S. Ozenne B. Armand S. (2022). Psilocybin-induced mystical-type experiences are related to persisting positive effects: A quantitative and qualitative report. *Front. Pharmacol.* 13:841648. 10.3389/fphar.2022.841648 35355714 PMC8959755

[B56] Millière R. (2017). Looking for the self: Phenomenology, neurophysiology and philosophical significance of drug-induced ego dissolution. *Front. Hum. Neurosci.* 11:245. 10.3389/fnhum.2017.00245 28588463 PMC5441112

[B57] Millière R. Carhart-Harris R. L. Roseman L. Trautwein F. M. Berkovich-Ohana A. (2018). Psychedelics, meditation, and self-consciousness. *Front. Psychol.* 9:1475. 10.3389/fpsyg.2018.01475 30245648 PMC6137697

[B58] Moreno F. A. Wiegand C. B. Keolani Taitano E. Delgado P. L. (2006). Safety, tolerability, and efficacy of psilocybin in 9 patients with obsessive-compulsive disorder. *J. Clin. Psychiatry* 67 1735–1740. 10.4088/JCP.v67n1110 17196053

[B59] Muthukumaraswamy S. D. Forsyth A. Lumley T. (2021). Blinding and expectancy confounds in psychedelic randomized controlled trials. *Expert Rev. Clin. Pharmacol.* 14 1133–1152. 10.1080/17512433.2021.1933434 34038314

[B60] Nichols D. E. (2016). Psychedelics. *Pharmacol. Rev.* 68 264–355. 10.1124/pr.115.011478 26841800 PMC4813425

[B61] Pahnke W. (1963). *Drugs and mysticism: An analysis of the relationship between psychedelic drugs and the mystical consciousness. Thesis presented to the President and Fellows of Harvard University for the Ph.D. thesis in Religion and Society.* Cambridge, MA: Harvard University.

[B62] Preller K. H. Vollenweider F. X. (2018). “Phenomenology, structure, and dynamic of psychedelic states,” in *Behavioral Neurobiology of Psychedelic Drugs. Current Topics in Behavioral Neurosciences*, eds Halberstadt A. L. Vollenweider F. X. Nichols D. E. (Berlin: Springer). 10.1007/7854_2016_459

[B63] Qiu T. T. Minda J. P. (2022). Psychedelic experiences and mindfulness are associated with improved wellbeing. *J. Psychoactive Drugs* 1–11. 10.1080/02791072.2022.2060773 35438609

[B64] Roseman L. Nutt D. J. Carhart-Harris R. L. (2018). Quality of acute psychedelic experience predicts therapeutic efficacy of psilocybin for treatment-resistant depression. *Front. Pharmacol.* 8:974. 10.3389/fphar.2017.00974 29387009 PMC5776504

[B65] Ross S. Bossis A. Guss J. Agin-Liebes G. Malone T. Cohen B. (2016). Rapid and sustained symptom reduction following psilocybin treatment for anxiety and depression in patients with life-threatening cancer: A randomized controlled trial. *J. Psychopharmacol.* 30 1165–1180. 10.1177/0269881116675512 27909164 PMC5367551

[B66] Sanders-Bush E. Breeding M. Knoth K. Tsutsumi M. (1989). Sertraline-induced desensitization of the serotonin 5HT-2 receptor transmembrane sig- naling system. *Psychopharmacology (Berl.)* 99 64–69. 10.1007/BF00634454 2550988

[B67] Segal Z. Williams J. Teasdale J. (2002). *Mindfulness- based cognitive therapy for depression: A new approach to preventing relapse.* New York, NY: Guilford.

[B68] Shapiro S. L. Brown K. W. Thoresen C. Plante T. G. (2011). The moderation of Mindfulness-based stress reduction effects by trait mindfulness: Results from a randomized controlled trial. *J. Clin. Psychol.* 67 267–277. 10.1002/jclp.20761 21254055

[B69] Sheehan D. V. Lecrubier Y. Sheehan H. K. Amorim P. Janavs J. Weiller E. (1998). The Mini-International Neuropsychiatric Interview (M.I.N.I.): The development and validation of a structured diagnostic psychiatric interview for DSM-IV and ICD-10. *J. Clin. Psychiatry* 59 22–33. 9881538

[B70] Sloshower J. Guss J. Krause R. Wallace R. M. Williams M. T. Reed S. (2020). Psilocybin-assisted therapy of major depressive disorder using Acceptance and Commitment Therapy as a therapeutic frame. *J. Context. Behav. Sci.* 15 12–19. 10.1016/j.jcbs.2019.11.002

[B71] Smigielski L. Kometer M. Scheidegger M. Krähenmann R. Huber T. Vollenweider F. X. (2019a). Characterization and prediction of acute and sustained response to psychedelic psilocybin in a mindfulness group retreat. *Sci. Rep.* 9:14914. 10.1038/s41598-019-50612-3 31649304 PMC6813317

[B72] Smigielski L. Scheidegger M. Kometer M. Vollenweider F. X. (2019b). Psilocybin-assisted mindfulness training modulates self-consciousness and brain default mode network connectivity with lasting effects. *Neuroimage* 196 207–215. 10.1016/j.neuroimage.2019.04.009 30965131

[B73] Soler J. Elices M. Franquesa A. Barker S. Friedlander P. Feilding A. (2016). Exploring the therapeutic potential of Ayahuasca: Acute intake increases mindfulness-related capacities. *Psychopharmacology (Berl.)* 233 823–829. 10.1007/s00213-015-4162-0 26612618

[B74] Spies M. Nasser A. Ozenne B. Jensen P. S. Knudsen G. M. Fisher P. M. (2020). Common HTR2A variants and 5-HTTLPR are not associated with human in vivo serotonin 2A receptor levels. *Hum. Brain Mapp.* 41 4518–4528. 10.1002/hbm.25138 32697408 PMC7555071

[B75] Stace W. (1960). *The teachings of the mystics.* New York, NY: New American Library.

[B76] Stenbæk D. S. Madsen M. K. Ozenne B. Kristiansen S. Burmester D. Erritzoe D. (2020). Brain serotonin 2A receptor binding predicts subjective temporal and mystical effects of psilocybin in healthy humans. *J. Psychopharmacol.* 35 459–468. 10.1177/0269881120959609 33501857

[B77] Studerus E. Gamma A. Kometer M. Vollenweider F. X. (2012). Prediction of psilocybin response in healthy volunteers. *PLoS One* 7:e30800. 10.1371/journal.pone.0030800 22363492 PMC3281871

[B78] Svarer C. Madsen K. Hasselbach S. G. Pinborg L. H. Haughbøl S. Frøkjaer V. G. (2005). MR-based automatic delineation of volumes of interest in human brain PET images using probability maps. *Neuroimage* 24 969–979. 10.1016/j.neuroimage.2004.10.017 15670674

[B79] Uthaug M. V. Lancelotta R. van Oorsouw K. Kuypers K. P. C. Mason N. Rak J. (2019). A single inhalation of vapor from dried toad secretion containing 5-methoxy-N,N-dimethyltryptamine (5-MeO-DMT) in a naturalistic setting is related to sustained enhancement of satisfaction with life, mindfulness-related capacities, and a decrement of psyc. *Psychopharmacology (Berl.)* 236 2653–2666. 10.1007/s00213-019-05236-w 30982127 PMC6695371

[B80] Uthaug M. V. van Oorsouw K. Kuypers K. P. C. van Boxtel M. Broers N. J. Mason N. L. (2018). Sub-acute and long-term effects of ayahuasca on affect and cognitive thinking style and their association with ego dissolution. *Psychopharmacology (Berl.)* 235 2979–2989. 10.1007/s00213-018-4988-3 30105399 PMC6182612

[B81] Vollenweider F. X. Vollenweider-Scherpenhuyzen M. F. I. Bäbler A. Vogel H. Hell D. (1998). Psilocybin induces schizophrenia-like psychosis in humans via a serotonin-2 agonist action. *Neuroreport* 9 3897–3902. 10.1097/00001756-199812010-00024 9875725

[B82] Way B. M. Creswell J. D. Eisenberger N. I. Lieberman M. D. (2010). Dispositional mindfulness and depressive symptomatology: Correlations with limbic and self-referential neural activity during rest. *Emotion* 10 12–24. 10.1037/a0018312 20141298 PMC2868367

[B83] Yatham L. N. Liddle P. F. Dennie J. Shiah I.-S. S. Adam M. J. A. Lane C. J. L. (1999). Decrease in Brain Serotonin 2 Receptor Binding in Patients With Major Depression Following Desipramine Treatment A Positron Emission Tomography Study With Fluorine-18–Labeled Setoperone. *Arch Gen Psychiatry* 56 705–711. 10.1001/archpsyc.56.8.705 10435604

