# Supplementary material for: Lasting increases in trait mindfulness after psilocybin correlate positively with the mystical-type experience in healthy individuals
Source: Front Psychol. 2022 Oct 5;13:948729. doi: 10.3389/fpsyg.2022.948729 (PMC9580465; doi:10.3389/fpsyg.2022.948729)
Supplement: Supplementary file 1 [file Table_1.DOCX]

**
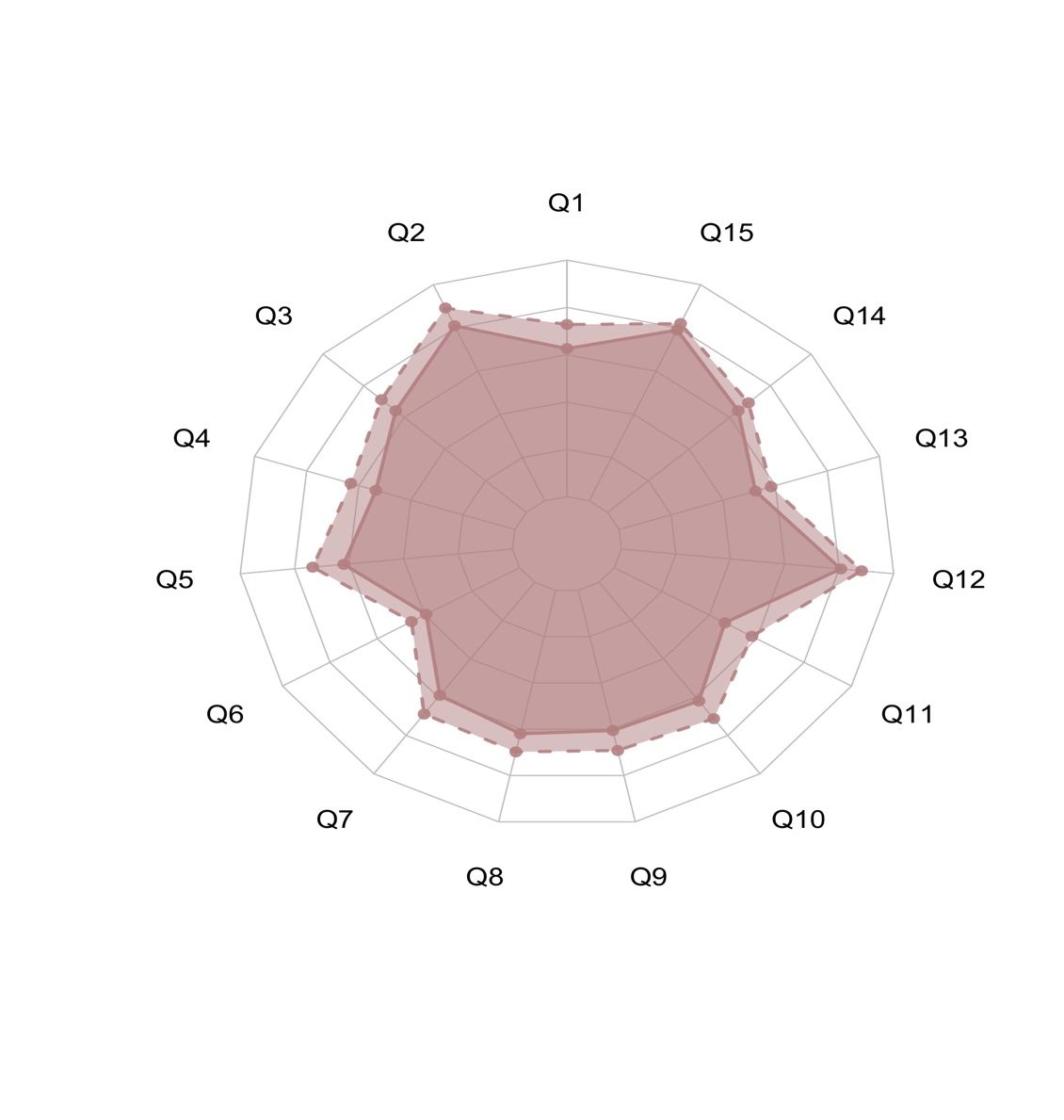
**

**Figure S1.** **Change across each mean item score in the MAAS.** There is a global increase from baseline to follow-up in all 15 MAAS items. We refer to the text version of the MAAS for the individual items (Brown & Ryan, 2003). Q = item. Full line: MAAS mean item score at baseline; Dashed line: MAAS mean item score at three-month follow-up. Grey lines in radar represent each score 1-6.

**
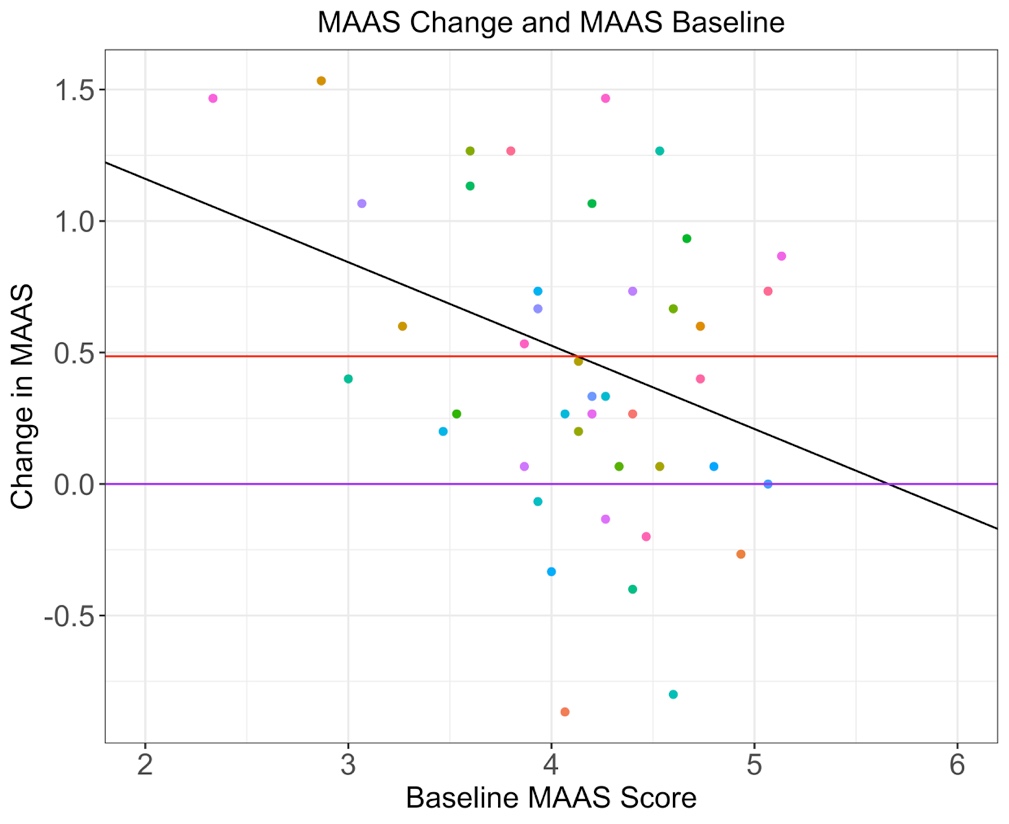
**

**Figure S1. Baseline MAAS and change in MAAS.** We here graphically illustrate each individual participant’s change in MAAS score against their baseline score, set in perspective to the mean score. Black line: estimated regression line. Red line: Mean change in MAAS score. Purple line: No change in MAAS score. Coloured circles: individual participants (same colour=participants with two interventions). No participants had a total MAAS score < 2.
